# Regional Disparities and Emerging Topics in Human Milk Research Across Africa: A Scoping Review

**DOI:** 10.1002/fsn3.70810

**Published:** 2025-08-15

**Authors:** Mustafa Mousa Basha, Linda P. Siziba, Rihab Omer Hamid, Jon Genuneit

**Affiliations:** ^1^ Zentrum für Klinische Studien (ZKS) Leipzig, Medical Faculty Leipzig University Leipzig Germany; ^2^ Pediatric Epidemiology, Department of Pediatrics, Medical Faculty Leipzig University Leipzig Germany

**Keywords:** Africa, breast milk, human milk, lactation, public health, scoping review

## Abstract

This scoping review explores the regional distribution and scope of human milk research in Africa. Given the nutritional and immunological importance of human milk, understanding regional research activity is crucial for addressing maternal and infant health challenges across the continent. A systematic search of the Web of Science database was conducted up to 10/2023 adhering to PRISMA‐ScR guidelines. All relevant publications on human milk research in Africa were included, regardless of type or quality. Studies were categorized based on key characteristics including study design, geographic location, and focus areas. A total of 459 reports on human milk sampling were identified, published between 1952 and 2023, with 81% full text available. Research activity was concentrated in Southern, Eastern, and Western Africa, with South Africa, Kenya, and Nigeria leading in HIV, micronutrient, and immunological research. Descriptive studies accounted for 34% of publications. HIV‐related research (21.6%) predominated in regions with high HIV prevalence. There was notable underrepresentation from Northern and Central Africa, with countries like Niger and Gabon showing limited activity. Micronutrient and pesticide research prevailed in South Africa, Kenya, and Nigeria, while human milk microbiota research was concentrated in South Africa and Kenya. This scoping review reveals significant regional disparities in human milk research across Africa. Southern and Eastern Africa lead in key areas, but Northern and Central Africa remain underrepresented. To address these gaps, increased collaboration, funding, and region‐specific research are essential. Expanding research efforts will enhance understanding and improve breastfeeding practices and infant health across the continent.

## Graphical abstract

Our scoping review analysed 459 reports published between 1952 and 2023, and thus identified regional imbalances in research output and thematic focus. Notably, Southern, Eastern, and Western Africa dominate the landscape, while Northern and Central Africa remain underrepresented. The findings underscore the need for targeted funding and collaboration to bridge these gaps and improve breastfeeding practices and infant health outcomes.

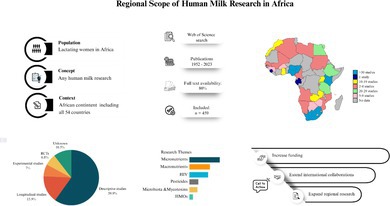

## 1 Introduction

Human milk is the optimal source of nutrition for infants, playing a pivotal role in promoting health, development, and survival (Victora et al. 2016). It contains a complex array of bioactive components that support immune maturation, organ development, and healthy microbial colonization, thus lowering the risks of infection, malnutrition, and mortality (Bode et al. 2020; Christian et al. 2021). Notably, research into human milk components has become increasingly pertinent on a global scale (Bode et al. 2020). However, precise data on human milk composition, including optimal ranges across different geographic regions and ethnicities, is still scarce (Christian et al. 2021). This disparity not only underscores a critical research gap but also raises questions about the contextual relevance and applicability of findings predominantly derived from high‐income countries.

Research on human milk has made significant strides in Africa, particularly in assessing its composition and understanding its role in improving infant survival and development (Horta et al. 2023; Rollins et al. 2016; Victora et al. 2016). In addition, studies have shown that breastfeeding practices in Africa are influenced by a variety of factors, including maternal nutrition, food insecurity, and cultural practices (Gutierrez‐de‐Terán‐Moreno et al. 2022). Yet, many regions in Africa face challenges like malnutrition, limited healthcare access, and suboptimal breastfeeding practices, which contribute to poor health outcomes for both infants and mothers (Pérez‐Escamilla et al. 2023). Thus, strengthening the implementation and enforcement of existing policies, along with raising awareness, could significantly reduce preventable maternal and child deaths (Victora et al. 2016).

Furthermore, understanding the composition of human milk in African populations is essential to designing interventions that address regional nutritional deficits and infectious disease burdens, thereby improving maternal and child health outcomes on the continent. We therefore sought to provide a comprehensive overview of the available studies in Africa that sampled human milk, to identify the current state of knowledge, and to highlight possible research gaps. By addressing regional disparities and highlighting emerging topics, this work contributes to bridging the gap between global research priorities and local needs, ensuring that the benefits of human milk are fully realized for African populations.

## 2 Methodology

### 2.1 Study Design

This scoping review was conducted following the Preferred Reporting Items for Systematic reviews and Meta‐Analyses—Scoping Reviews (PRISMA‐ScR) guideline (Tricco et al. 2018) to describe the current knowledge and identify gaps in human milk research in Africa. To ensure methodological clarity and transparency, we structured our inclusion criteria using the Population‐Concept‐Context (PCC) framework recommended for scoping reviews (Pollock et al. 2023):

- *Population*: Studies that investigated human milk samples collected at any lactation stage from women in any African country.
- *Concept*: Research focusing on human milk composition, properties, and core health implications including macronutrients, micronutrients, microbiota, and infectious diseases.
- *Context*: The geographical scope was limited to the African continent, including all 54 countries and historical/regional terms such as “Rhodesia” or “Sahara.”

### 2.2 Information Sources and Search Strategy

The search was last updated on October 1, 2023, using the Web of Science library database, which is available on subscription through Leipzig University. This included databases such as MEDLINE, BIOSIS, Grants Index, KCI‐Korean Journal Database, Preprint Citation Index, ProQuest Dissertation & Theses, Scientific Electronic Library Online (SciELO), Science Citation Index Expanded, Social Sciences Citation Index, Emerging Sources Citation Index, and Arts and Humanities Citation Index. Of note, although Web of Science includes a broad selection of global and regional databases (e.g., SciELO, MEDLINE), it does not comprehensively index African Journals Online (AJOL) or many locally published African journals. As such, some regionally specific studies that may not be indexed internationally may not have been captured, resulting in a limited representation of the literature retrieved. This is a recognized limitation, and the results should be interpreted with this potential bias in mind.

The search strategy was designed to maximize inclusivity. The search terms included variants of “milk”, “lactation”, “breastfeeding”, “humanmilk”, “colostrum”, “breastfed”, and the German term “Muttermilch”, connected with the Boolean operator “OR” to form a first block. The second block contained all 54 countries and older geopolitical names (e.g., “Rhodesia”), regional terms (“Sahara”), and general terms like “afric”*; also including their English, French, and German spellings, also connected by the Boolean operator “OR” within the block. These two blocks were then joined with the Boolean operator “AND”, and this combined query was searched within titles, abstracts, and author keywords with no restrictions on publication dates. Given the broad and overarching nature of the review, the authors chose not to perform a meta‐analysis.

### 2.3 Eligibility Criteria

Studies were included in this scoping review if they: (i) were conducted in any African country, (ii) sampled human milk at any lactation stage, (iii) were published in English, German, and French (languages in which MMB has proficiency). All studies that did not meet the inclusion criteria were excluded.

## 3 Data Extraction and Analysis

The publications retrieved from the search were imported into Covidence systematic review software (Veritas Health Innovation, Melbourne, Australia) in RIS format, and duplicates were automatically removed.

### 3.1 Screening

In the first round, titles and abstracts were independently screened by two co‐authors (MMB and ROH) for eligibility. When discrepancies arose, the two co‐authors (MMB and ROH) first discussed the conflict among themselves. If a consensus could not be reached, the other two co‐authors (LPS and JG) were consulted for a final decision, after which all conflicts were resolved through consensus discussions involving all co‐authors. This multi‐step process was done to ensure consistency, accuracy, and minimize individual bias.

### 3.2 Data Extraction

In the second round, data was extracted following a standardized protocol agreed upon by all co‐authors. Extracted information included key study characteristics such as first author, year of publication, country of study, study design, sample size, and study aims (e.g., HIV, milk composition, lactation stage ≤ 6 months or > 6 months, microbiota). Study aims were further categorized into the following thematic areas: macronutrients (e.g., Human milk oligosaccharides [HMOs], amino acids [AA], and fatty acids [FAs]); micronutrients (e.g., minerals, vitamins, and metals/trace elements); microbiota (bacteria, fungi, and viruses—excluding HIV).

### 3.3 Data Analysis

Descriptive analysis was done using SPSS version 29.0 (IBM Corporation, Somers, NY, USA) and R (version 3.5.1; R Foundation for Statistical Computing, Vienna, Austria). The Mann‐Kendall test was used to test for a monotonic trend of how research focus has evolved over time. Data analysis was finalized in December 2024, as the process required careful organization and validation of the extensive data collected up to October 2023.

## 4 Results

### 4.1 Overview

**FIGURE 1:**
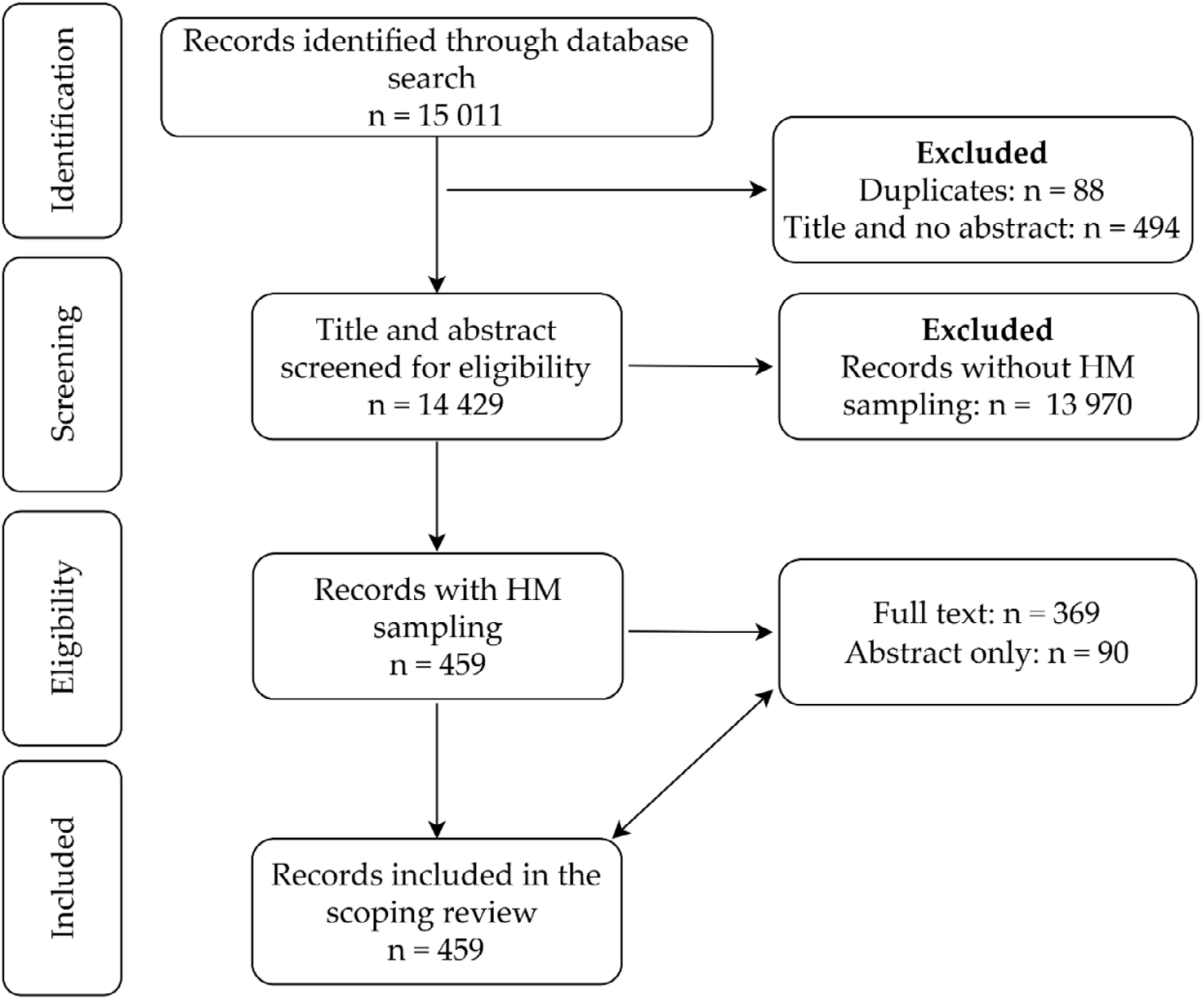
Flow diagram of study selection process. HM, human milk.

A total of 459 papers specifically involving human milk sampling done in Africa and published between 1952 and 2023 were included (Figure 1). Full texts were available for the majority (*n* = 369, 81%) of publications. About a third were descriptive studies (*n* = 146, 31.8%, Table 1).

**TABLE 1:** Categorization of included studies.

Category	Details	*n* (%)
Study design	Descriptive	275 (59.9)
	Longitudinal	73 (15.9)
	Experimental	32 (7)
	RCTs	31 (6.8)
Study focus	HIV	99 (21.6)
	Micronutrients	98 (21.4)
	Macronutrients	40 (8.7)
	Microbiota	27 (5.9)
Lactation stage	≤ 6 months	177 (38.6)
	> 6 months	7 (1.5)
	Unknown	275 (59.9)

*Note:* Frequencies may not add up to 100 because of missings, that is, the information on the respective categories may not have been stated in the manuscript. Percentages were calculated using *n* = 459.

Abbreviations: HIV, Human Immunodeficiency Virus; RCT, randomized controlled trial.

### 4.2 Population: Study Characteristics and Sampling

**FIGURE 2:**
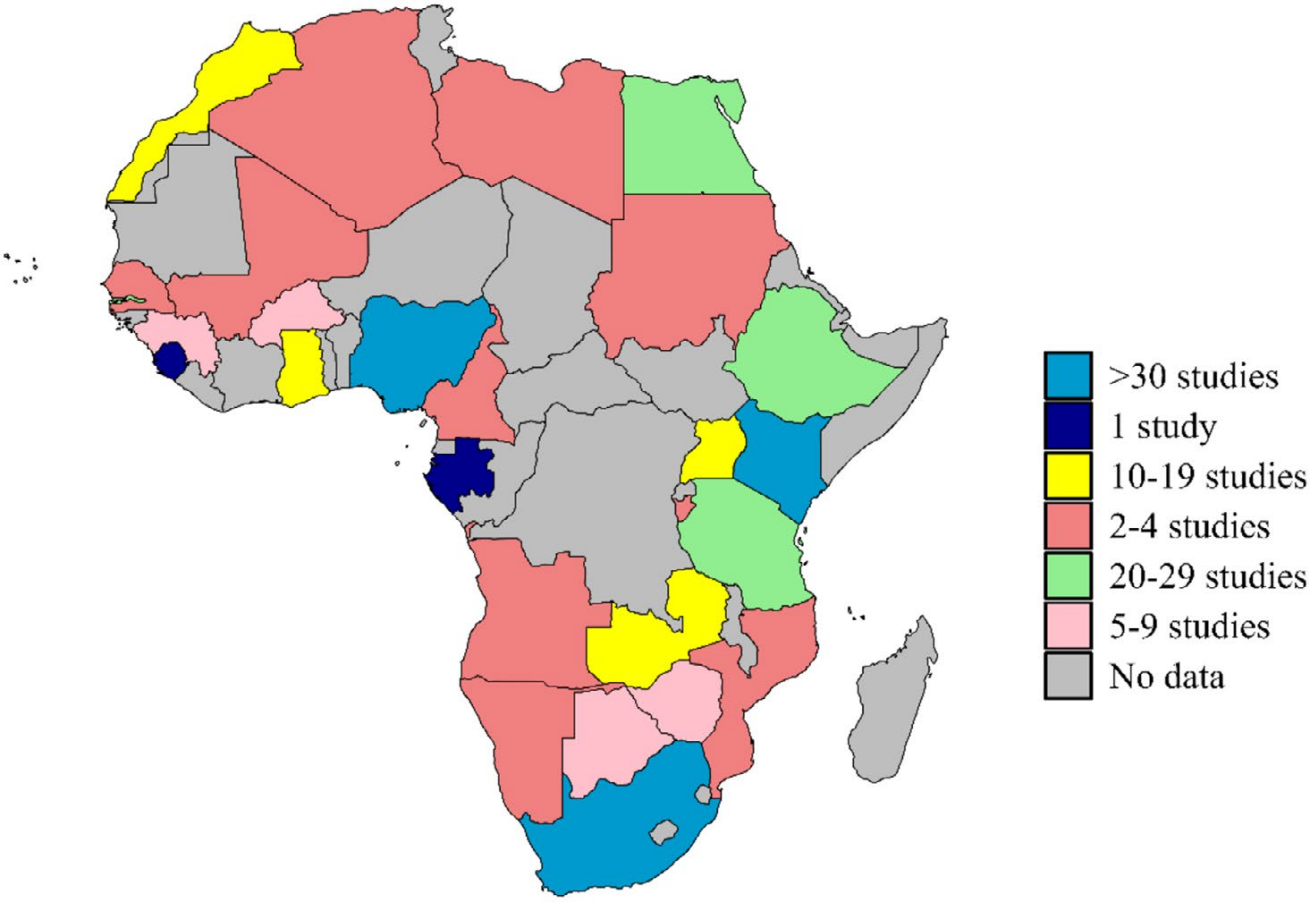
Number of research studies done in Africa by country.

Lactation stage was explicitly reported in approximately half of the studies (*n* = 184, 40.1%), and about a third (*n* = 177, 38.6%) involved samples collected early in lactation (i.e., < 6 months postpartum, Table 1). Countries were categorized based on the United Nations geoscheme for Africa, which distinguishes five major regions: North, West, East, Central, and Southern Africa (Table 2). The geographical spread of research across Africa was uneven (Figure 2), with the highest research activity in East Africa (*n* = 132, 28.7%) and Southern Africa (*n* = 131, 28.5%), followed by West Africa (*n* = 115, 25%), North Africa (*n* = 50, 10.9%), and Central Africa (*n* = 31, 6.8%, Table 2). A few countries (e.g., Niger, Sao Tome, Sierra Leone, and Gabon) had minimal or no research on human milk (Figure 2).

**TABLE 2:** Research focus on human milk composition across African regions.

Research focus	Western Africa (*n* = 115)	Southern Africa (*n* = 131)	Eastern Africa (*n* = 132)	Central Africa (*n* = 31)	Northern Africa (*n* = 50)
Macronutrients/milk intake	24 (20.9)	23 (17.6)	19 (14.4)	9 (29.0)	5 (10.0)
HMO	4 (3.5)	10 (7.6)	4 (3.0)	2 (6.5)	1 (2.0)
Fatty acids	9 (7.8)	6 (4.6)	8 (6.1)	2 (6.5)	0
Micronutrients	27 (23.5)	27 (20.6)	23 (17.4)	5 (16.1)	10 (20.0)
Calcium	10 (8.7)	3 (2.3)	4 (3.0)	3 (9.7)	1 (2.0)
Zinc	10 (8.7)	3 (2.3)	5 (3.8)	0	5 (10.0)
Iodine	2 (1.7)	3 (2.3)	1 (0.8)	0	0
HIV research	7 (6.1)	48 (36.6)	40 (30.3)	3 (9.7)	0
Microbiota & mycotoxins	13 (11.3)	13 (9.9)	9 (6.8)	5 (16.1)	6 (12.0)
Pesticides	9 (7.8)	16 (12.2)	9 (6.8)	1 (3.2)	14 (28.0)

*Note:* Values are *n* (%).

Abbreviations: HIV, human immunodeficiency virus; HMO, Human milk oligosaccharides.

### 4.3 Concept: Thematic Areas of Research

**FIGURE 3:**
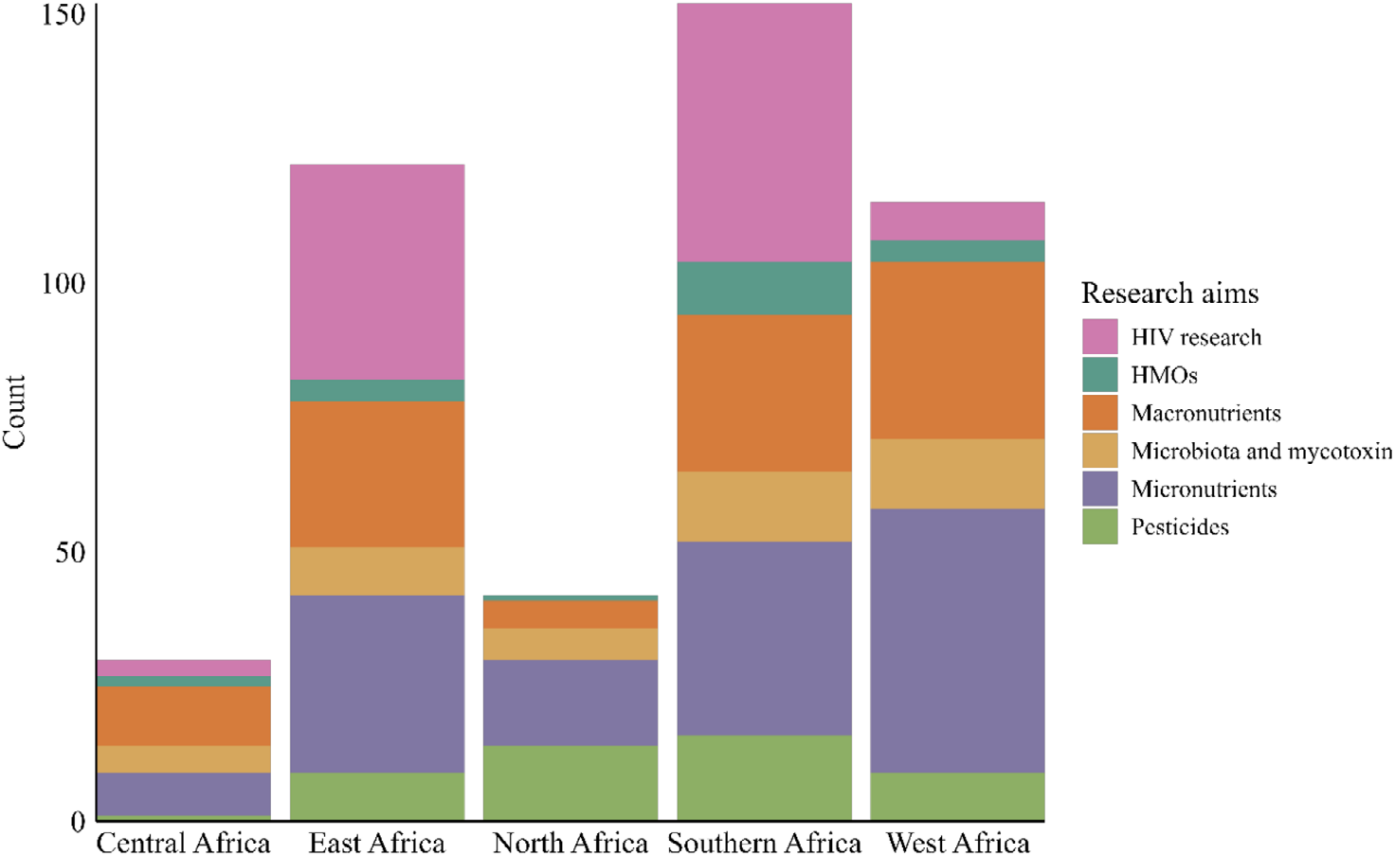
Stacked bar chart represents research focus across African regions. The numbers do not add up to the total number of publications included because there was an overlap for the countries in which some of the studies were conducted.

The included studies covered a wide range of thematic areas, with HIV being the most prominent topic accounting for 21.6% (*n* = 99) of all publications (Table 1). These studies were primarily concentrated in South Africa, Kenya, and Zambia, likely reflecting both the regional disease prevalence and associated funding priorities (Table 3). Micronutrient research was similarly prevalent (*n* = 98, 21.4%), with a focus on essential nutrients such as zinc, iron, and Vitamin A (Table 3). Studies investigating macronutrients were less common (*n* = 40, 8.7%) and were primarily in West and Southern Africa (24 and 23 studies, respectively, Table 2). Research on HMOs was geographically concentrated in Southern Africa (*n* = 10 studies, Table 2 and Figure 3), while calcium and zinc studies were most frequently conducted in West Africa (Table 2). Microbiota and mycotoxin‐related research accounted for 5.9% (*n* = 27) of the total and was largely done in South Africa, Nigeria, and Kenya, with minimal studies in North and Central Africa (Figure 3). Immunological components, such as lactoferrin and IgA, were predominantly studied in South Africa, Kenya, and Ethiopia. However, studies exploring cytokines and less commonly examined immune molecules remain limited. Environmental exposures, particularly pesticide contamination, were most frequently reported in South Africa (*n* = 11), followed by Egypt (*n* = 7) and Tunisia (*n* = 4). COVID‐19 antibodies in human milk represented an emerging research area but were only captured in a single study from Uganda (Table 3), indicating a gap in response to recent global health events.

**TABLE 3:** Summary of research on human milk research across African countries.

Country	Studies (*n*)	Research focus	Collaborations
Algeria	3	Minerals, trace elements	
Angola	2	Vitamins, microbiological indicators	Ghana
Botswana	5	Vitamins (C), minerals, HIV	
Burkina Faso	5	Trace elements, HIV, immunological components (IGF1, S100B)	Europe, Côte d'Ivoire, Ethiopia
Burundi	3	Cu, Fe, Zn, Se, vitamins, microbiological indicators	Europe, Congo
Cameroon	3	Minerals, protozoa (malaria), vitamins (A)	
Central Africa	10	Na, Ca, vitamins, HIV, pesticides, microbiological indicators	South Africa, Europe, Asia, America
Egypt	25	Minerals (Zn, Pb, Ca), vitamins (A, E, C), pesticides, microbiological indicators	USA, African regional research
Ethiopia	20	Ca, Zn, I, Fe, vitamins (A), HIV, pesticides, microbiological indicators, immunological components	Europe, America, South Africa, Kenya, Gambia, Ghana
Gabon	1	Minerals	
Gambia	26	Ca, Zn, Se, vitamins (B12), HIV, pesticides, microbiological indicators, immunological components (IGF1, TNFα)	South Africa, Kenya, Ethiopia, Namibia, Ghana, Nigeria, Europe, Asia, America
Ghana	12	Pb, As, Cd, Hg, vitamins (A), HIV, pesticides, immunological components	Europe, America, South Africa, Kenya, Gambia, Ethiopia
Guinea	8	Ca, Na, K, Ebola, pesticides	America, Guinea‐Bissau
Kenya	48	HIV, HMO, AA, Ca, Fl, Zn, Fe, vitamins (A, B, Folate, B12), EBV, CMV, pesticides, microbiological indicators	Europe, America, South Africa, Uganda, Gambia, Ethiopia, Ghana, Zimbabwe
Libya	2	Zn, Fe, Cu, Se	
Mali	2	Trace elements, HIV	
Morocco	12	Zn, Pb, Cd, Hg, vitamins (A), cortisol, viruses, pesticides	Asia, Europe
Mozambique	4	Minerals, vitamins, HIV	South Africa, Gambia, Europe, Asia, America
Namibia	4	Minerals, vitamins, microbiological indicators, pesticides	
Nigeria	48	Trace elements (Se, Ca, Na, Zn, Fe, I, Cu, Pb, etc.), vitamins (A, Folate), viruses (measles, malaria), etc.	Europe, Asia, Uganda, Kenya, Gambia, Zimbabwe, Congo
Senegal	4	Vitamins, minerals, microbiological indicators, viruses, pesticides	
Seychelles	1	Trace elements	
Sierra Leone	1	Trace elements, other indicators	
South Africa	79	HIV, micronutrients, macronutrients, immunological components, mycotoxins, microbiota, pesticides, etc.	Broad geographical, international (14 studies)
Sudan	3	Minerals, trace elements, HIV	Europe, America
Tanzania	24	Na, I, Hg, vitamins (B12), HIV, pesticides, immunological components (RANTES, SLPI)	Europe, Asia, America, South Africa, Zimbabwe, Zambia
Uganda	18	Ca, P, Mg, Na, protozoa (malaria), HIV, pesticides	South Africa, regional collaborations
Zambia	18	Pb, Na, K, Hg, vitamins (A), HIV, immunological components (RANTES, CRP)	South Africa, Uganda, Tanzania
Zimbabwe	9	Zn, Hg, vitamins, HIV, pesticides	Kenya, Tanzania, Nigeria

Abbreviations: AA, Amino Acids; Ca, Calcium; CMV, Cytomegalovirus; CRP, C‐reactive Protein; Cu, Copper; EBV, Epstein–Barr Virus; Fe, Iron; Fl, Fluoride; Hg, Mercury; HIV, Human Immunodeficiency Virus; HMO, Human Milk Oligosaccharides; IGF1, Insulin‐like Growth Factor 1; K, Potassium; Mg, Magnesium; Na, Sodium; Normal T‐cell Expressed and Secreted; P, Phosphorus; Pb, Lead; RANTES, Regulated on Activation; Se, Selenium; SLPI, Secretory Leukocyte Protease Inhibitor; TNFα, Tumor Necrosis Factor Alpha; Zn, Zinc.

### 4.4 Context: Regional Trends and Research Networks

Overall, the research landscape across Africa demonstrates a diverse research activity (Figure 3). Southern Africa led in overall research output (*n* = 79 studies, Table 3), particularly on HIV, micronutrients, and immunological components. Kenya and Nigeria, each with 48 studies, had a broader spread of research themes including environmental contaminants and immunological markers. West Africa focused heavily on micronutrients and fatty acids, particularly calcium and zinc. North and Central Africa showed sparse research activity overall, highlighting geographic disparities in research funding and infrastructure.

### 4.5 Collaboration Networks

Collaboration patterns suggest strong international partnerships, especially involving institutions from Europe and America (Table 3), which potentially influence both research funding and study design. Countries with stronger research infrastructure (e.g., South Africa, Kenya, and Nigeria) were more frequently involved in multi‐country studies. Smaller countries (e.g., Mozambique, Senegal, and Namibia) participated in fewer studies but were often included in internationally led projects, highlighting external research dependency.

### 4.6 Trends Over Time

An analysis of temporal trends showed a statistically significant growth in research activity (Figure S1, *p* = 0.01) and research themes (*p* = 0.001) over time. Research in the early years (1952–1979) was limited, with a primary focus on macronutrients and micronutrients (Figure S2). There was a notable increase in publications in the micronutrients category between 1980 and 1990. This was followed by a significant rise in research output across nearly all themes, particularly HIV and macronutrients, which had the highest number of publications. Research on microbiota and mycotoxins, as well as pesticides, seemed to remain relatively stable.

### 4.7 Publication Bias

While a wide range of topics and regions are represented in this scoping review, the predominance of descriptive studies and the reliance on international funding and publishing platforms likely contribute to publication bias. Research that is conducted locally or relevant to specific regions, especially from under‐resourced areas, may remain unpublished or appear in journals that are not indexed in Web of Science. This further skews the thematic and geographic representation of research in Africa in this dataset.

## 5 Discussion

This scoping review offers a comprehensive overview of human milk research conducted in African countries from 1952 to 2023. Using the PCC framework, we examined studies involving human milk from African women (population), focusing on themes such as composition, health implications, and environmental exposures (concept), within the diverse geographical and socio‐political contexts of African regions (context). Our findings show an uneven distribution of research across the continent, with Southern, Eastern, and parts of Western Africa leading in research activity, while Central and North Africa remain underrepresented. There is a clear growth in research activity over time, with peaks in certain themes such as HIV and micronutrients, followed by a mix of trends over time. This suggests shifting research priorities and possibly the maturation of some fields.

### 5.1 Population: Disparities in Geographic and Research Capacity

The concentration of research in South Africa, Kenya, and Nigeria not only reflects the public health priorities of these countries (e.g., high HIV prevalence) but also the presence of relatively well‐established research infrastructure and international collaborations. For instance, South Africa's dominant role in HIV‐related research is closely linked to its high disease burden (Rautenbach et al. 2024; Zuma et al. 2022) and strong network of research institutions and funders. In contrast, Central and North African countries seemingly lack consistent funding pipelines and institutional frameworks to support large‐scale studies, thereby limiting their contribution to the regional knowledge base on human milk. Notably, these differences in national research capacity directly influence the amount of studies, diversity of research themes, and collaborative patterns in the region. As such, countries with supposedly limited infrastructure may appear more in projects led externally as opposed to being independent contributors. All this highlights the systematic inequities in research representation. Addressing these gaps will require long‐term investment in local research capacity, including independent training, funding, and institutional support.

### 5.2 Concept: Thematic Areas and Research Gaps

The diversity of research themes identified in this scoping review highlights the public health concerns that are evolving in Africa. On one hand, HIV‐related studies were the most predominant research theme, particularly in Southern and Eastern Africa. This is possibly driven by the high prevalence of the HIV burden in these regions (Parker et al. 2021; Vrazo et al. 2018; World Health Organisation (WHO) 2023) as well as public health collaborations. On the other hand, there is a growing interest in COVID‐19 antibodies in human milk. Yet, only one study was identified, and this area of research remains underexplored even in these regions. In addition, the main focus of nutritional research was on micronutrients, including zinc, selenium, and calcium, as well as trace elements, particularly in Nigeria, Kenya, and Egypt. These findings highlight a growing interest in maternal diet and its implications for infant health (Carretero‐Krug et al. 2024), while also showing a lack of data from regions like Central Africa that are ecologically diverse but also underrepresented.

Furthermore, environmental contaminants, such as mycotoxins and pesticides, can be transferred to the infant through human milk, thus affecting its safety (Ojo‐Okunola et al. 2018; Olisah et al. 2020), but are also a growing threat to food security (Nji et al. 2022; Onyeaka et al. 2024). Human milk research makes it possible to assess this exposure to both the mother and the infant, but this research theme was concentrated in Northern and Southern Africa. Further studies are therefore needed to investigate the pathways of exposure and associated risks across different ecological and socio‐economic environments, particularly among these underrepresented regions.

Moreover, immunological studies which focus on components like IgA and lactoferrin are increasingly showing potential for informing disease prevention strategies in infants (Davis et al. 2022; Pammi and Gautham 2020). Despite this, there are still some knowledge gaps with regard to the role of some immunological markers (e.g., IgM, IgG, and cytokines) in health and disease (Jones et al. 2020; Santaolalla et al. 2021). This is especially true in countries that are not major centers of scientific research, thus highlighting the geographic and scientific knowledge gaps in this region.

### 5.3 Context: Cultural, Socioeconomic, and Health System Influences

Breastfeeding practices and research priorities are significantly influenced by different and region‐specific cultural norms (Amzat et al. 2024). For instance, in some North African contexts, complementary food is introduced earlier (Kavle et al. 2014), colostrum is perceived as harmful (Odeniyi et al. 2020), and other socio‐religious practices limit breastfeeding in public (Odeniyi et al. 2020). All these may reduce both the duration of exclusive breastfeeding and overall interest in conducting in‐depth studies on human milk. Conversely, in regions where the prevalence of HIV is high, such as Southern and Eastern Africa (World Health Organisation (WHO) 2023), breastfeeding is not only a source of nutrition but also a means of disease prevention and transmission (Taha et al. 2024; Vrazo et al. 2018), thereby shaping the direction of research that focuses on the immunological properties of human milk in these regions.

Furthermore, the variability in healthcare systems also shapes research priorities. On one hand, countries that have relatively strong maternal and child health systems in place, such as Kenya and Ethiopia (Melesse et al. 2024; Zhu et al. 2024) are better positioned to support longitudinal and advanced biomarker analysis in human milk research. These systems often benefit from consistent governmental funding and support or longstanding international collaborations. On the other hand, countries with relatively weaker healthcare systems and infrastructure may not have access to established ethical review boards, essential tools needed for surveillance, or laboratory capacity and resources. As a result, it becomes difficult to conduct reliable human milk research due to these methodological constraints.

Socioeconomic disparities further add to these challenges. For instance, in many parts of West Africa, environmental pollution from electronic waste processing, mining, and inadequate sanitation practices can lead to the accumulation of toxins in human milk (Abogunrin‐Olafisoye and Adeyi 2025; Olujimi et al. 2023). Similarly, high levels of food insecurity, which are primarily driven by climate change, conflict, or economic stability, also affect maternal nutrition and subsequently human milk composition (Bottin et al. 2022). The contrast between urban and rural environments adds another layer to it, in that mothers in urban areas may have different eating patterns, experience more breastfeeding challenges associated with employment, and even access different health systems compared to mothers in rural areas (Adhikari et al. 2022). Understanding all these contextual differences is important for interpreting these results and the heterogeneity of the focus of human milk studies across the African continent. This is also important for designing meaningful studies and implementing effective and culturally sensitive public health and nutrition interventions. Still, extending this research to other African regions could help address disparities in food safety and dietary practices.

### 5.4 Policy Implications

Our findings highlight the need to incorporate human milk research into wider maternal and child health initiatives throughout Africa. In addition, funding for research on nutritional adequacy, food safety, and prevention of infectious diseases through human milk should be a priority. Third, more data on contaminants such as pesticides and mycotoxins is needed to inform environmental health policies and food regulation networks. Lastly, more research on immune components is needed to guide the development of breastfeeding guidelines that are region‐specific, particularly in areas with a high burden of infectious disease.

### 5.5 Future Directions and Recommendations

To foster progress and address the current research and knowledge gaps, it is important to expand research capacity to regions that are currently underrepresented. This can be possible with dedicated funding schemes, developing local training programs, and investing in infrastructure. In addition, fostering equitable collaborations that empower African researchers to lead in projects and define research priorities would in turn improve the relevance and sustainability of research efforts. Thematic expansion is further needed to address understudied areas such as cytokine profiling, COVID‐19 antibodies in human milk, and the effect of environmental exposures specific to different ecologic regions. Finally, incorporating human milk research into national health policy frameworks could strengthen links between nutrition, infectious diseases, and environmental health, thereby promoting a more holistic approach to maternal and child health across the continent.

A major limitation of this scoping review is that the literature search ended on October 1, 2023, meaning any studies published after this date were not included. Much like any systematic attempt to synthesize existing evidence, this restricts the review's ability to incorporate the latest developments in the field. Nevertheless, the extensive scope of included studies provides a comprehensive and robust overview of the research landscape up to that point. Another limitation is the exclusive use of the Web of Science database for the search, though this encompasses MEDLINE and many other sources. While expanding the search to additional databases such as SCOPUS, Embase, or CINAHL could have yielded more studies, many African journals are not comprehensively indexed in these databases either (Asubiaro 2023). Manual searches, though potentially more inclusive, are resource‐intensive and would have been in contrast to the objective of quickly providing an overview of the field in this scoping review. Despite all this, the number of publications included suggests that any missed studies are unlikely to significantly alter the overarching conclusions of this review.

This review included all relevant studies, irrespective of their type or quality, aligning with the aim of scoping reviews to provide an overview of existing literature rather than synthesize evidence (Pham et al. 2014). Consequently, we did not conduct a formal assessment of the methodological quality of individual studies, which is an acknowledged limitation of scoping reviews. Additionally, the predominance of descriptive studies in this field may introduce publication bias, as positive findings are more likely to be published, potentially skewing the representation of the available evidence.

## 6 Conclusion and Implications for Future Research

In conclusion, this scoping review highlights evident disparities in human milk research across Africa, potentially resulting from differences in research infrastructure, public health priorities, and cultural context. Applying the PCC framework systematically highlighted the importance of addressing not only what is studied (concept), but also who is studied (population), and where (context). Thus, future efforts should target underrepresented regions and underexplored research themes in order to improve breastfeeding outcomes and infant health. Cross‐sector collaboration, culturally informed approaches, and equitable funding schemes are also essential to building a more inclusive and impactful evidence base.

## Author Contributions

Conceptualisation: M.M.B., L.P.S., R.O.H., and J.G. Data acquisition: M.M.B. Analysis: M.M.B., L.P.S. Interpretation: M.M.B., L.P.S., R.O.H., and J.G. Drafting of the manuscript: M.M.B., L.P.S., and J.G. Revision and final approval of the manuscript: all authors.

## Ethics Statement

The authors have nothing to report.

## Conflicts of Interest

J.G. is the project manager and L.P.S. is a scientist on unrestricted research grants from Danone Nutricia Research to Ulm and Leipzig University for research into human milk and lactation with the Ulm SPATZ Health Study. This work is not related to the current publication.

## Supporting information


**Figure S1:** Number of publications and research activity between 1952 and 2023.
**Figure S2:** Trends in the main research topics over time across different decades between 1952 and 2023.

## Data Availability

All data are available upon request from jon.genuneit@medizin.uni‐leipzig.de.
