# Supplementary material for: Regional Disparities and Emerging Topics in Human Milk Research Across Africa: A Scoping Review
Source: Food Sci Nutr. 2025 Aug 15;13(8):e70810. doi: 10.1002/fsn3.70810 (PMC12355963; doi:10.1002/fsn3.70810)
Supplement: Supplementary file 1 — Figure S1: Number of publications and research activity between 1952 and 2023. Figure S2: Trends in the main research topics over time across different decades between 1952 and 2023. [file FSN3-13-e70810-s001.docx]

**Regional disparities and emerging topics in human milk research across Africa: a scoping review**

Mustafa Mousa Basha^1,*^, Linda P. Siziba^2*^, Rihab Omer Hamid^2^, Jon Genuneit^2^

^1^Zentrum für Klinische Studien (ZKS) Leipzig, Medical Faculty, Leipzig University, Leipzig, Germany

^2^Pediatric Epidemiology, Department of Pediatrics, Medical Faculty, Leipzig University, Leipzig, Germany

^*^Authors contributed equally

Correspondence:

Linda.Siziba@medizin.uni-leipzig.de

Pediatric Epidemiology

Department of Pediatrics

Medical Faculty, Leipzig University

Liebigstr. 20a, Haus 6

04103 Leipzig

Germany

+49 341 97 24181

**Supplementary material**


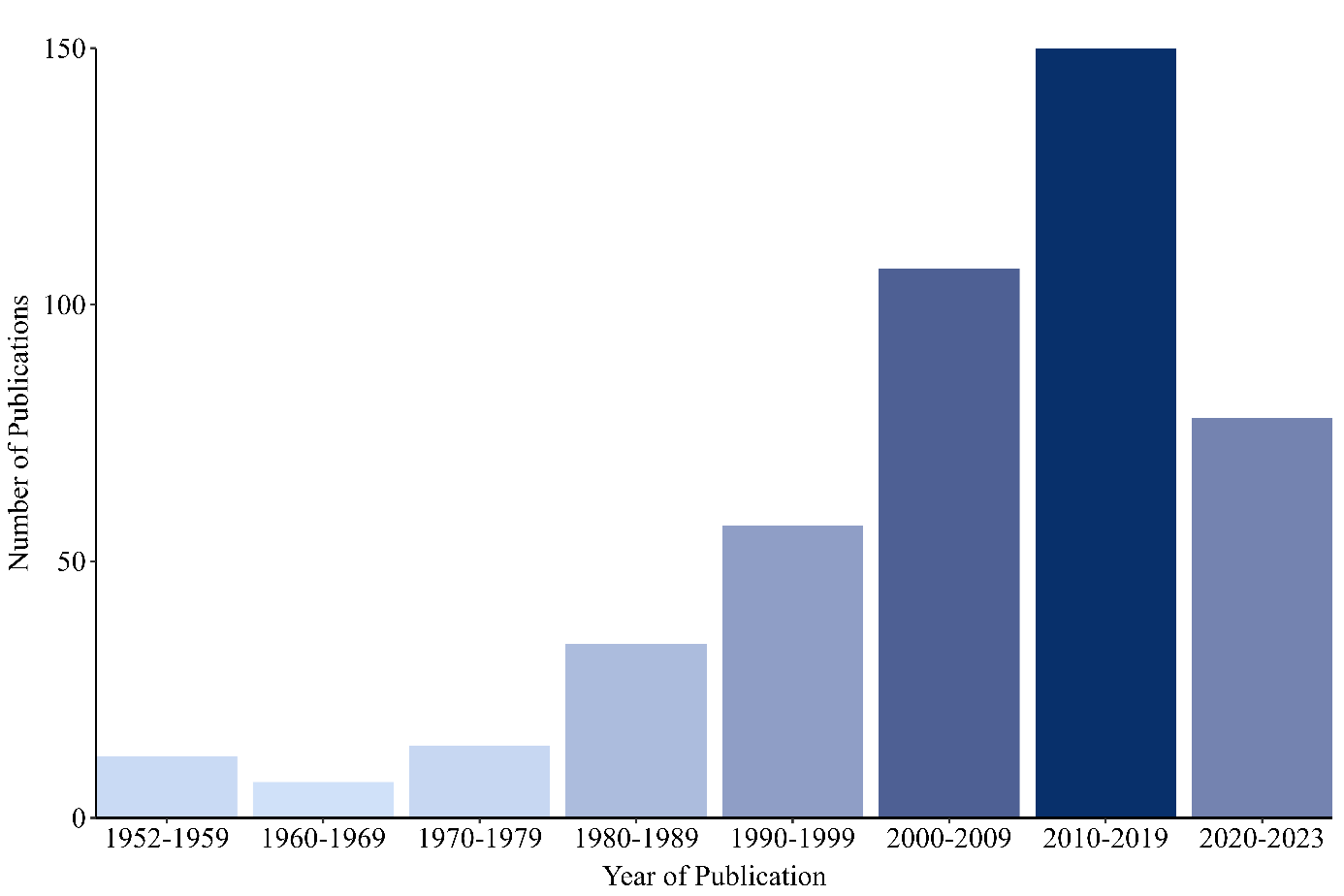


**Figure S1**: Number of publications and research activity between 1952 and 2023.


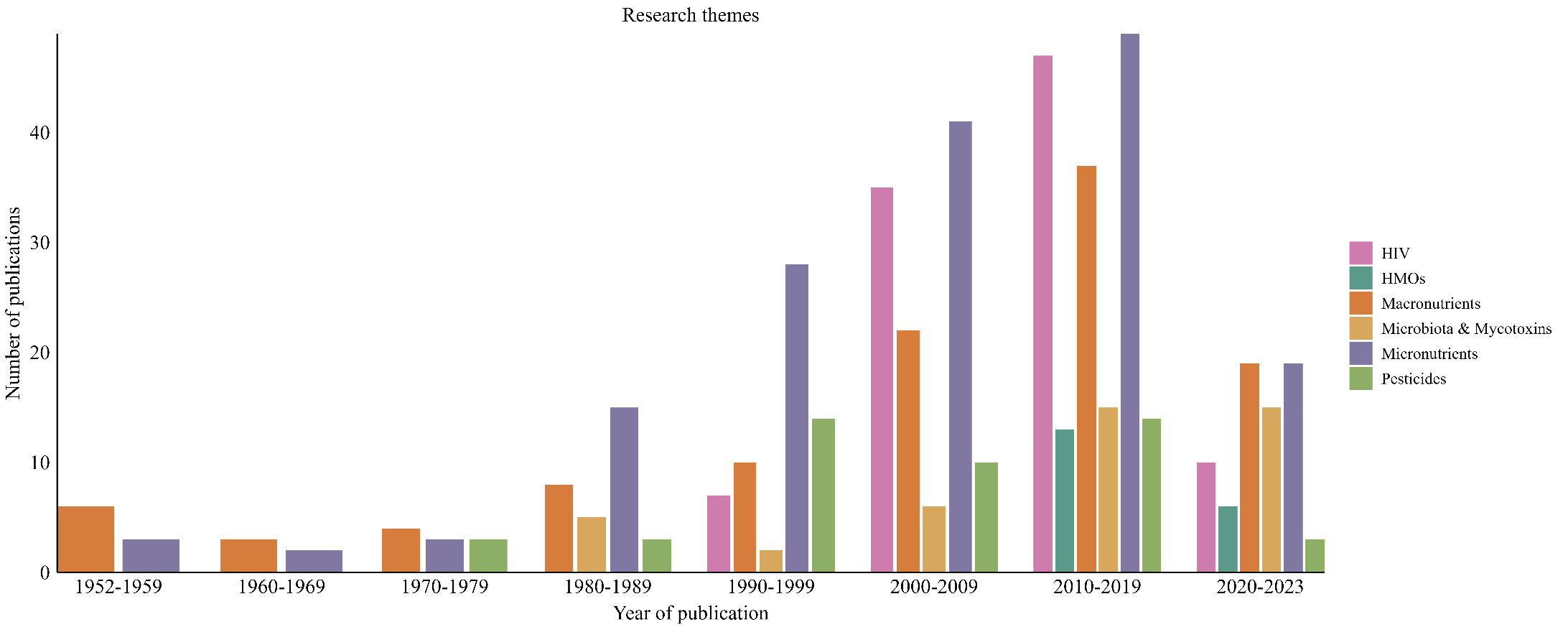


**Figure S2**: Trends in the main research topics over time across different decades between 1952 and 2023.
